# Galanin-immunoreactivity identifies a distinct population of inhibitory interneurons in laminae I-III of the rat spinal cord

**DOI:** 10.1186/1744-8069-7-36

**Published:** 2011-05-15

**Authors:** Sheena YX Tiong, Erika Polgár, Josie C van Kralingen, Masahiko Watanabe, Andrew J Todd

**Affiliations:** 1 Institute of Neuroscience and Psychology, College of Medical, Veterinary and Life Sciences, University of Glasgow, Glasgow G12 8QQ UK; 2 Department of Anatomy, Hokkaido University School of Medicine, Sapporo 060-8638, Japan

## Abstract

**Background:**

Inhibitory interneurons constitute 30-40% of neurons in laminae I-III and have an important anti-nociceptive role. However, because of the difficulty in classifying them we know little about their organisation. Previous studies have identified 3 non-overlapping groups of inhibitory interneuron, which contain neuropeptide Y (NPY), neuronal nitric oxide synthase (nNOS) or parvalbumin, and have shown that these differ in postsynaptic targets. Some inhibitory interneurons contain galanin and the first aim of this study was to determine whether these form a different population from those containing NPY, nNOS or parvalbumin. We also estimated the proportion of neurons and GABAergic axons that contain galanin in laminae I-III.

**Results:**

Galanin cells were concentrated in laminae I-IIo, with few in laminae IIi-III. Galanin showed minimal co-localisation with NPY, nNOS or parvalbumin in laminae I-II, but most galanin-containing cells in lamina III were nNOS-positive. Galanin cells constituted ~7%, 3% and 2% of all neurons in laminae I, II and III, and we estimate that this corresponds to 26%, 10% and 5% of the GABAergic neurons in these laminae. However, galanin was only found in ~6% of GABAergic boutons in laminae I-IIo, and ~1% of those in laminae IIi-III.

**Conclusions:**

These results show that galanin, NPY, nNOS and parvalbumin can be used to define four distinct neurochemical populations of inhibitory interneurons. Together with results of a recent study, they suggest that the galanin and NPY populations account for around half of the inhibitory interneurons in lamina I and a quarter of those in lamina II.

## Background

Inhibitory interneurons constitute around one third of the neurons in laminae I-II of rat dorsal horn, and ~40% of those in lamina III [1]. Immunocytochemical studies suggest that virtually all of these are GABAergic, with some using glycine as a co-transmitter [1,2]. GABAergic and glycinergic inhibition in the dorsal horn has an important antinociceptive role [3,4], and its loss is thought to contribute to chronic pain states [5-7]. Sandkuhler [7] has identified four separate functions for inhibitory interneurons in this region: (1) regulating the level of activity of nociceptive projection neurons in order to ensure an appropriate response to noxious stimuli, (2) preventing spontaneous activity in these cells in the absence of noxious stimuli, (3) minimising cross-talk between sensory modalities (e.g. between low-threshold mechanoreceptive and nociceptive inputs to dorsal horn neurons), and (4) limiting the spatial spread of activity to somatotopically appropriate regions of the dorsal horn. It is likely that these different functions are performed by distinct populations of inhibitory interneurons, each with specific patterns of synaptic input and output. However, despite their importance, we still know little about how inhibitory interneurons are organised into different functional populations, and how these are incorporated into the synaptic circuitry of the dorsal horn [8].

There have been many attempts to classify interneurons in this region based on morphological and/or electrophysiological criteria [9-22]. Lamina II has been extensively studied, and the most widely accepted scheme is that of Grudt and Perl [16], who identified four main morphological classes: islet, central, vertical and radial cells. Recent electrophysiological studies have found that these classes account for 70-80% of neurons recorded in this lamina [11,16,18,19]. It has also been shown that there is a relationship between morphology and neurotransmitter type, since all islet cells are inhibitory, while radial and most vertical cells are excitatory [17,19,23]. However, those inhibitory neurons that are not islet cells are morphologically diverse, and include vertical and central cells, as well as neurons that cannot be assigned to any of these classes. Even less is known about functional populations of interneurons in laminae I and III.

An alternative approach to classifying interneurons is based on differential expression of neurochemical markers [24]. For example, certain neuropeptides, such as neuropeptide Y (NPY) and galanin, are expressed by inhibitory interneurons, while others (somatostatin, neurotensin, neurokinin B) are found in excitatory cells [25-30]. In addition, the calcium-binding protein parvalbumin and the neuronal isoform of nitric oxide synthase (nNOS) are expressed by some inhibitory neurons in laminae I-III [31-34]. We have previously demonstrated that among the inhibitory interneurons, NPY, nNOS and parvalbumin are present in non-overlapping populations [32]. We have also shown that these differ in their postsynaptic targets, since axons that contain NPY and GABA preferentially innervate large projection neurons in lamina III that express the neurokinin 1 receptor (NK1r) [35,36], while nNOS-immunoreactive GABAergic axons selectively innervate giant lamina I projection cells that lack the NK1r [37]. Little is apparently known about the postsynaptic targets of the axons of parvalbumin-containing interneurons, although these are thought to include the central terminals of low-threshold mechanoreceptive myelinated primary afferents, with which they form axoaxonic synapses (DI Hughes and BA Graham, personal communication).

Galanin is expressed by many peptidergic primary afferents in the dorsal horn [38,39], but is also present in some GABAergic neurons in laminae I-III [26]. Galanin-containing cells may therefore form a further population of inhibitory interneurons, distinct from those that contain NPY, nNOS or parvalbumin. The first aim of this study was to test this hypothesis by looking for co-localisation of these compounds in neuronal cell bodies. We went on to provide quantitative data for the galanin interneuron population, by determining the proportion of neurons and of GABAergic axons in each lamina that were galanin-immunoreactive.

## Methods

### Animals

Eight adult male Wistar rats (Harlan, Loughborough, UK; 230 - 310 g) were deeply anaesthetised with pentobarbitone and perfused through the left ventricle with Ringer's solution followed by 4% freshly depolymerised formaldehyde. Lumbar spinal cord segments (L2-L4) were removed, stored in the same fixative for 5-24 hours and then cut into 60 μm thick transverse sections with a vibrating microtome. All experiments were approved by the Ethical Review Process Applications Panel of the University of Glasgow, and were performed in accordance with the European Community directive 86/609/EC and the UK Animals (Scientific Procedures) Act 1986. All efforts were made to minimize the number of animals used and their suffering.

For all of the immunocytochemical reactions described in subsequent sections, incubations in primary and secondary antibodies were carried out at 4°C. Species-specific secondary antibodies raised in donkey were obtained from Jackson Immunoresearch, West Grove, PA, USA (conjugated to biotin, Rhodamine Red, Cy5 or DyLight 649) or Invitrogen, Paisley, UK (conjugated to Alexa 488). Those conjugated to biotin, Alexa 488 or DyLight 649 were used at 1:500, while those conjugated to Rhodamine Red or Cy5 were used at 1:100. In all cases, apart from those that involved tyramide signal amplification (TSA), the antibodies were diluted in phosphate buffered saline that contained 0.3M NaCl but no blocking serum. TSA reactions, which were used to ensure optimal detection of galanin in the cell bodies of neurons, were performed using a tetramethylrhodamine kit (PerkinElmer Life Sciences, Boston, MA, USA) according to the manufacturer's instructions. The selection of sections for analysis in each part of the study was made before immunofluorescence staining was viewed.

### Test for co-localisation of galanin with parvalbumin or nNOS

Sections from the L4 segments of 3 rats were incubated for 3 days in a mixture of rabbit anti-galanin (Peninsula/Bachem, St Helens, UK; 1:20,000), affinity-purified guinea pig antibody against parvalbumin [40] (1.08 μg/ml) and sheep anti-nNOS [41] (1:2,000), and then overnight in secondary antibodies conjugated to biotin (anti-rabbit IgG), Cy5 and Alexa 488. The galanin was revealed with a TSA reaction, as described above. Two sections from each rat were scanned sequentially (to avoid fluorescent bleedthrough) with a Bio-Rad Radiance 2100 confocal microscope, equipped with Argon, green HeNe and red diode lasers, through a 40× oil-immersion lens. Because of the limited area covered by the objective lens, it was necessary to obtain 6 or 7 z-series from each section. These were scanned at 2 μm z-separation through the full thickness of the section. Scans were analysed with Neurolucida for Confocal software (MicroBrightField Inc, Colchester, VT, USA) and the locations of all galanin- and parvalbumin-containing neurons were drawn onto an outline of the dorsal horn. Each galanin cell was examined to determine whether it was also immunoreactive for parvalbumin or nNOS, while the parvalbumin cells were examined to test whether they were nNOS-positive.

### Test for co-localisation of galanin with NPY

Sections from the L2 segments of 3 rats were incubated for 4 days in rabbit anti-galanin (1:50,000) and overnight in biotinylated anti-rabbit IgG, followed by detection with a TSA reaction. They were then incubated for 2 days in rabbit anti-NPY (Bachem; 1:500) and mouse monoclonal antibody NeuN (Millipore, Watford, UK; 1:1000), and overnight in secondary antibodies: anti-rabbit IgG conjugated to Alexa 488 and anti-mouse IgG conjugated to Cy5. Note that although both galanin and NPY antibodies were raised in rabbit, the concentration of galanin antibody was too low for it to be detected by the Alexa 488-labelled secondary antibody. Two sections from each animal were scanned with the confocal microscope as described in the previous section. The scans were analysed with Neurolucida for Confocal and the locations of all neurons that were immunoreactive for galanin or NPY were indicated on drawings of the dorsal horn. Each of these neurons was examined to determine whether it showed one or both types of neuropeptide immunoreactivity. In order to look for evidence of co-localisation of peptide immunoreactivity in axonal boutons, 100 NPY-immunoreactive boutons were selected in confocal scans from a single section for each of the 3 rats. The selection of boutons was made before the galanin immunostaining was viewed, and boutons were sampled throughout the full thickness of laminae I-III. Each of the selected boutons was then examined to determine whether it was also galanin-immunoreactive.

### Test for co-localisation of NPY and nNOS

We have previously reported that NPY was not co-localised with reduced nicotinamide adenine dinucleotide phosphate (NADPH) diaphorase activity (a marker for nNOS) in laminae I-III [32]. However, it is possible that the NADPH diaphorase reaction failed to detect some nNOS-containing neurons, and we therefore tested for co-localisation of NPY and nNOS. Sections from the L3 segments of 3 rats were incubated for 3 days in rabbit anti-NPY (1:500) and sheep anti-nNOS (1:1000), and then overnight in appropriate secondary antibodies conjugated to Alexa 488 and Rhodamine Red. Two sections from each rat were scanned with the confocal microscope and the scans were analysed with Neurolucida for Confocal as described above. The locations of all NPY-immunoreactive cells were plotted onto a drawing of the dorsal horn, and each was then examined to determine whether it was nNOS-positive.

### Proportion of neurons in laminae I-III that contain galanin

Transverse sections from the L3 segments of 3 rats were incubated for 3 days in a mixture of rabbit anti-galanin (1:20,000) and NeuN (1:500), and then overnight in secondary antibodies: anti-mouse IgG conjugated to Cy5 and biotinylated anti-rabbit IgG. Galanin was revealed with a TSA reaction and the sections were then incubated in Sytox (Invitrogen; 1:50,000) for 30 mins at 20°C to reveal cell nuclei.

Two sections from each rat were scanned through a 40× oil-immersion lens with the confocal microscope, to produce sets of z-series that covered the entire cross-sectional area of laminae I-III, each consisting of 24 optical sections with 1 μm z-spacing. The scans were analysed with a modification [42] of the optical disector technique [43-46]. Merged images of NeuN and Sytox-staining were first viewed with Neurolucida for Confocal. In each z-series, the 14th optical section was designated as the reference section and the 22nd as the look-up section. Each optical section in the series was examined, and the locations of all neuronal nuclei (defined by the presence of both NeuN-immunoreactivity and Sytox) that were present in the reference section or appeared in subsequent sections in the series were plotted onto an outline of the grey matter. All of those cells with nuclei that were still present on the look-up section were then excluded, leaving only neurons for which the bottom surface of the nucleus was located between reference and look-up sections. The red channel (corresponding to galanin immunoreactivity) was then viewed and the presence or absence of galanin immunostaining in each of the selected neurons was determined. Boundaries between laminae I, II and III were identified from low-magnification scans obtained with light transmitted through a dark-field condenser [35,47], while the location of the ventral border of lamina III was determined from an atlas of rat spinal cord [48]. Laminar borders were plotted on the drawings, and in this way we were able to determine the proportions of neurons in laminae I, II and III that were galanin-immunoreactive. Since the number of lamina I neurons sampled in these sections was relatively small, we also scanned lamina I from a further 2 sections from each of the 3 rats. These were analysed in the same way, except that only cells in lamina I were included.

Although the reference and look-up sections are relatively far apart, we ensured that all neurons with nuclei that lay between these two planes were included in the sample by examining every optical section between them. The positions of reference and look-up sections within the z-series were arranged so that perikaryal cytoplasm at both poles of the nucleus was visible in all cases. This was done to ensure that galanin immunoreactivity could be detected even in weakly labelled neurons. No correction was made for tissue shrinkage, since our aim was to determine the proportion of neurons that were galanin-immunoreactive, rather than the absolute number of cells in a volume of tissue.

### Proportion of GABAergic boutons in laminae I-III that contain galanin

Antibody against the vesicular GABA transporter (VGAT) was used to identify GABAergic axons [35,49-51]. Transverse sections from the L3 segments of 3 rats were incubated for 3 days in a mixture of rabbit anti-galanin (1:1,000), monoclonal mouse antibody against VGAT (Synaptic Systems, Göttingen, Germany; 1:1,000) and guinea pig anti-calcitonin gene-related peptide (CGRP; Bachem; 1:10,000), and then overnight in secondary antibodies conjugated to Alexa 488, Rhodamine Red or Cy5.

Two sections from each of the 3 animals were scanned sequentially with the confocal microscope through a 60× oil-immersion lens with a z-separation of 0.3 μm. For each section, a set of 3 or 4 z-series (each containing at least 24 optical sections) was acquired in such a way as to cover a vertical strip through the central part of laminae I-III.

The scans were analysed with Neurolucida for Confocal. Initially, scans corresponding to VGAT-immunostaining were viewed, and those from the same section were aligned so that the full thickness of laminae I, II and III within the scanned strip was revealed. The laminar borders (identified as described above) were drawn onto an overlay, and a 5 × 5 μm grid was placed over the confocal image stacks. A single optical section (the 10th in the z-series) was viewed and 100 VGAT-immunoreactive boutons that were present in this section were selected from each lamina. This was done by selecting the VGAT boutons located nearest the bottom right corners of the grid squares. For each lamina, the first bouton was obtained from one of the most dorsal squares, and the selection process then continued with squares in a dorsal-to-ventral, followed by left-to-right, direction until 100 boutons had been acquired. Once the selection was completed, the files corresponding to galanin immunoreactivity were viewed and the presence or absence of galanin in each of the selected boutons was noted. Since this selection method will inevitably be biased towards boutons that were more extensive in the z-axis [45], we estimated the z-axis lengths of all of the selected VGAT-immunoreactive boutons by counting the number of optical sections (0.3 μm z-spacing) on which they appeared.

### Characterisation of antibodies

Details of the primary antibodies used in this study are given in Table 1. We have shown that immunostaining with the galanin and NPY antibodies in the dorsal horn can be abolished by pre-incubation with the corresponding peptide [26,28]. It has also been reported that neuronal staining with the galanin antibody is absent in the brains of galanin knock-out mice [52]. The nNOS antibody labels a band of 155 kDa in Western blots of rat hypothalamus, and immunostaining is abolished by pre-incubation in nNOS [41]. The parvalbumin antibody recognises a single band of 13 kDa in blots of mouse brain homogenates [40]. The NeuN antibody was generated against cell nuclei extracted from mouse brain and found to react with a protein specific to neurons [53]. We have shown that this antibody apparently labels all neurons (and no glial cells) in the rat spinal cord [47]. The mouse monoclonal VGAT antibody labels a single band of 57 kDa in blots of mouse brain and retina, and immunostaining is blocked by pre-absorption with the immunising peptide [54]. No staining with this antibody is seen in Western blots from cultured neurons obtained from VGAT^-/- ^mice [55]. The CGRP antibody detects both α and β forms of the peptide (manufacturer's specification).

**Table 1:** Primary antibodies

Antibody	Host	Antigen	Supplier/reference	Catalogue number	Dilution
Galanin	Rabbit polyclonal	rat galanin	Peninsula/Bachem	IHC 7141 (T-4334)	1:20-50,000 (TSA) 1:1,000

nNOS	Sheep polyclonal	rat nNOS	Herbison et al. [41]		1:1,000

Parvalbumin	Guinea pig polyclonal	mouse parvalbumin	Nakamura et al. [40]		1.08 μg/ml

NPY	Rabbit polyclonal	rat NPY	Bachem	T-4070	1:500

NeuN	Mouse monoclonal	Purified cell nuclei from mouse brain	Millipore	MAB377	1:500-1,000

VGAT	Mouse monoclonal	Amino acids 75-87 of rat VGAT coupled to KLH	Synaptic Systems	131 011	1:1,000

CGRP	Guinea pig polyclonal		Bachem	T-5027	1:10,000

## Results

### Distribution of galanin in the dorsal horn

**Figure 1:**
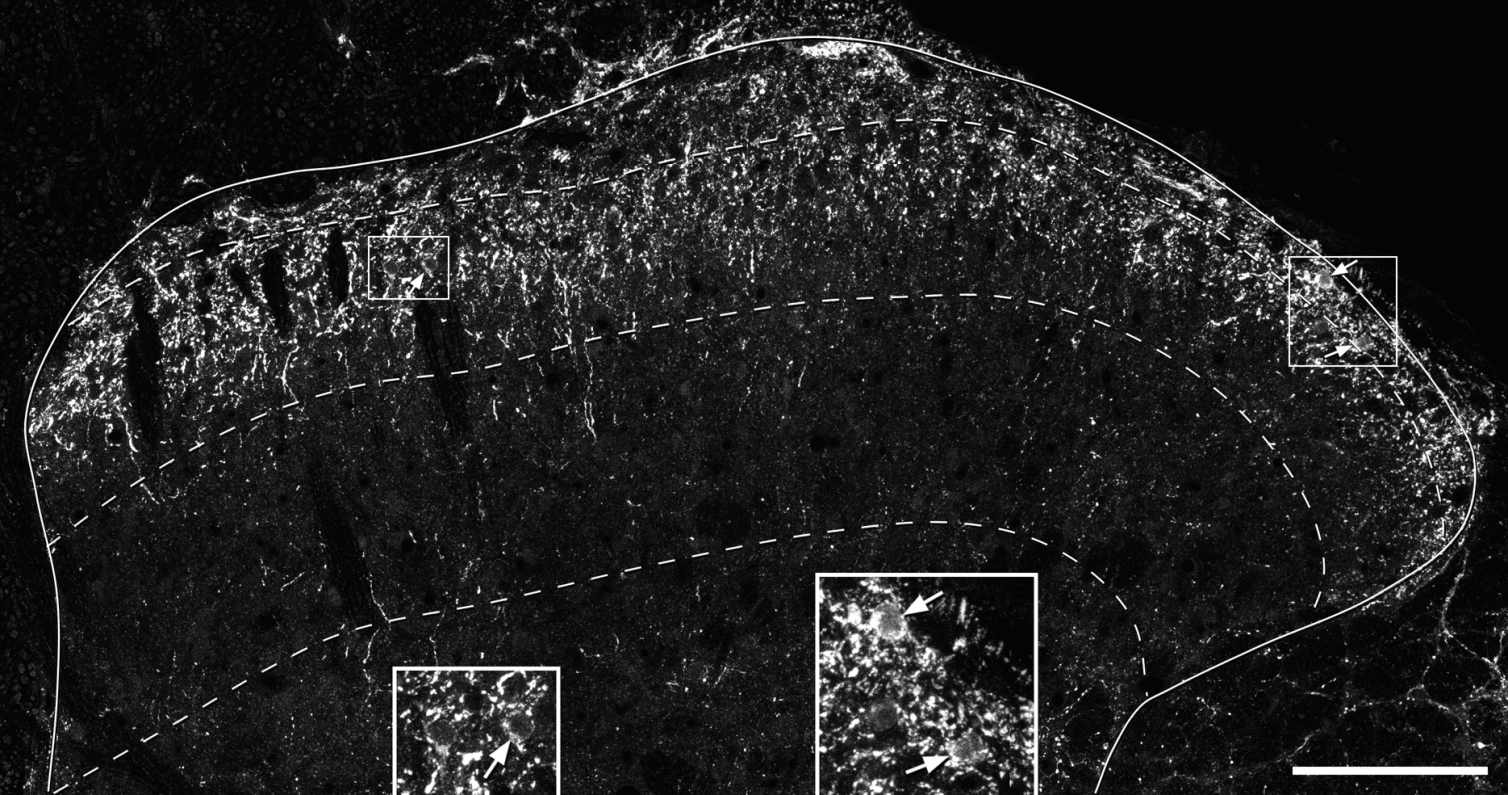
**Galanin immunoreactivity in laminae I-III**. A confocal image taken from a transverse section of the L3 segment that had been reacted to reveal galanin. The solid line represents the outer limit of the grey matter, and the dashed lines show the borders between laminae I, II and III. There is a dense plexus of immunoreactive axons in lamina I and the outer part of lamina II, with some staining in deeper parts of the dorsal horn. Scattered immunoreactive cell bodies are visible, and three of these are marked with arrows. The insets show the two regions outlined by boxes in the main figure, and have been enlarged to show the immunoreactive cell bodies more clearly. The image is a projection of 6 optical sections at 1 μm z-spacing. Scale bar = 100 μm.

The distribution of galanin-immunoreactivity was similar to that described in the rat dorsal horn in several previous studies [38,39,56-61]. Immunoreactive axons formed a dense plexus in the superficial region, particularly lamina I and the outer half of lamina II (IIo), while they were also seen at lower density in other laminae (Figure 1). Scattered immunoreactive cell bodies were observed, mainly in laminae I and IIo, but occasionally in the inner half of lamina II (IIi) and in lamina III. The immunostaining in these cells occupied the perikaryal cytoplasm, with limited extension into dendritic trees.

### Extent of co-localisation of galanin, NPY, nNOS and parvalbumin

**Figure 2:**
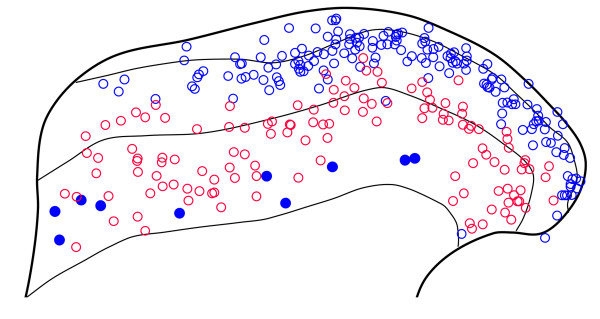
**The distribution of cells that were galanin or parvalbumin immunoreactive in sections reacted to reveal galanin, parvalbumin and nNOS**. The diagram shows the laminar location of all of the cells that were galanin (blue) or parvalbumin (red) immunoreactive in the 6 sections that were analysed (2 each from 3 rats). For the galanin cells, those that were also nNOS immunoreactive are shown as filled circles, while those that lacked nNOS are open circles. None of the parvalbumin cells contained nNOS, and there was no colocalisation of galanin and parvalbumin.

**Figure 3:**
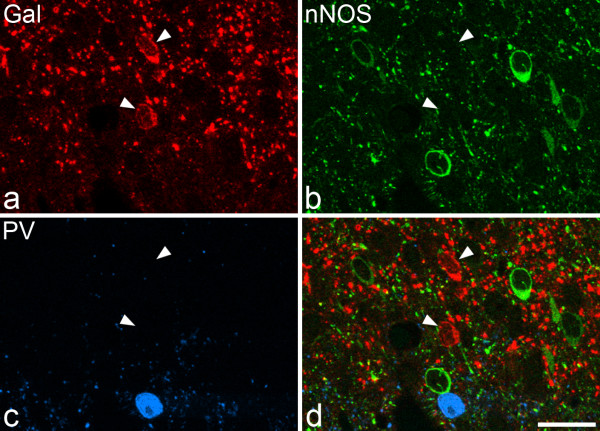
**Lack of co-localisation of galanin, nNOS and parvalbumin in lamina II**. **a **Confocal image showing part of lamina II that has been scanned to reveal galanin (Gal, red). Two galanin-containing cell bodies are visible (arrowheads). **b, c **The same field scanned for nNOS (green) and parvalbumin (PV, blue). In each case, immunoreactive cell bodies can be seen. **d **The merged image shows the lack of co-localisation of the three types of immunoreactivity in the cell bodies. The images were obtained from a single optical section. Scale bar = 20 μm.

**Figure 4:**
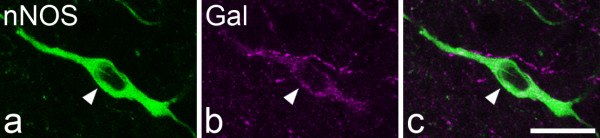
**Co-localisation of galanin and nNOS in a lamina III neuron**. **a **Confocal image shows a lamina III neuron that is immunoreactive for nNOS (green). **b **The same field scanned for galanin (Gal, magenta) shows that the same cell is weakly immunoreactive. **c **A merged image. The images are from three optical sections at 2 μm z-spacing. Scale bar = 20 μm.

One hundred and seventy four galanin-immunoreactive cells (48-63 per rat) were identified in the sections reacted to reveal galanin, parvalbumin and nNOS (Figures 2, 3). Although galanin-positive cells were found in each of laminae I-III, they were far more numerous in laminae I and IIo. Twelve of the cells were located in lamina III, and these all showed weak galanin immunoreactivity (Figure 4b). The pattern of immunostaining for parvalbumin was similar to that reported in previous studies [31,32,62], with scattered cell bodies and a plexus of dendrites and axons located on either side of the lamina II/III border. One hundred and twenty eight parvalbumin-immunoreactive cells (35-47 per rat) were identified in these sections. These were found in lamina II (mainly IIi) and lamina III. None of the galanin-immunoreactive cells was labelled by the parvalbumin antibody and *vice versa *(Figures 2, 3). The pattern of nNOS immunostaining was very similar to that of nNOS-immunoreactivity or NADPH diaphorase activity that has been reported in previous studies [32-34,63]. Many nNOS-immunoreactive cells were seen in lamina II, with a lower density in laminae I and III. These were far more numerous than either the galanin or parvalbumin cells, and therefore their locations were not included in Figure. 2. None of the parvalbumin cells, and none of the galanin cells in laminae I or II, were nNOS-immunoreactive (Figures 2, 3). However, 10 of the 12 galanin cells in lamina III were positive for nNOS (Figures 2, 4).

**Figure 5:**
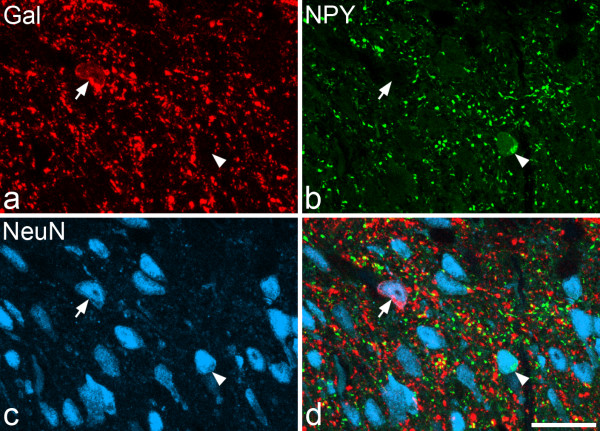
**Lack of co-localisation of galanin and NPY**. **a**, **b**, **c **show a confocal image from laminae I-IIo scanned for galanin (Gal, red), NPY (green) and the neuronal marker NeuN (blue), respectively. **d **A merged image. One of the NeuN-positive cell bodies is galanin-immuno-reactive (arrow) and another is NPY-immunoreactive (arrowhead). The image is a projection of 3 optical sections at 1 μm z-separation. Scale bar = 20 μm.

In the sections reacted to reveal galanin and NPY, the distribution of NPY staining was as described previously [35,64,65], with a dense axonal plexus in laminae I-II and scattered immunoreactive cells throughout laminae I-III. We identified 199 galanin-immunoreactive cells (52-86 per rat) and 251 NPY-immunoreactive cells (75-97 per rat) in these sections, and all of these were NeuN-positive, confirming that they were neurons. Within this sample, 1 cell (corresponding to 0.5% of the galanin population and 0.4% of the NPY population) located in lamina II showed both types of immunoreactivity, while all of the remaining cells were only labelled with antibodies against one of the peptides (Figure 5). Only 2 of the 300 NPY-immunoreactive boutons that had been selected (100 each from 3 rats) were galanin-immunoreactive.

**Figure 6:**
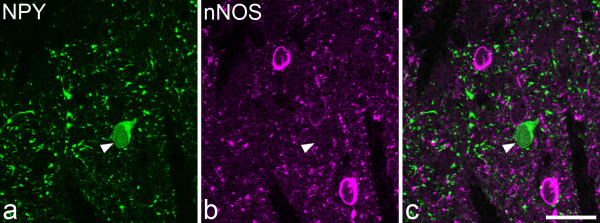
**Lack of co-localisation of NPY and nNOS**. A single confocal optical section through lamina II, scanned to reveal: **a **NPY (green) and **b **nNOS (magenta). A merged image is shown in **c**. This field contains cell bodies that are immunoreactive for NPY (arrowhead) or nNOS, but the two types of immunoreactivity are not co-localised. Scale bar = 20 μm.

Two hundred and twenty four NPY-immunoreactive cells (71-80 per rat) were found in the sections that had been reacted to reveal NPY and nNOS. Of these, 222 (99%) were not nNOS-immunoreactive, while two cells (one in lamina I and one in lamina II) were double-labelled. An example of a NPY cell that was negative for nNOS is illustrated in Figure 6.

### Proportion of neurons in laminae I-III that contain galanin

**Figure 7:**
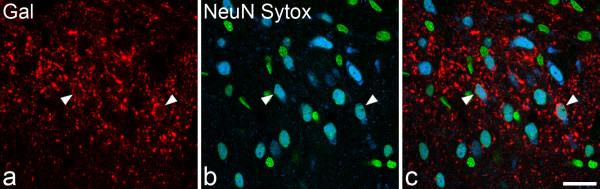
**Staining for galanin, NeuN and Sytox in a section used for stereological analysis**. **a **A single confocal optical section through laminae I and II scanned to reveal galanin (Gal, red). **b **The same field scanned for NeuN (blue), and the nuclear stain Sytox (green). **c **A merged image. Neuronal nuclei can be recognised by the presence of both NeuN and Sytox staining, and therefore appear cyan, while non-neuronal nuclei are green. Two of the neurons (arrowheads) are immunoreactive for galanin. This appears in a ring surrounding the nucleus, corresponding to the perikaryal cytoplasm. Scale bar = 20 μm.

The penetration of galanin immunostaining was apparently complete in the sections that had been reacted with anti-galanin, NeuN antibody and Sytox, since galanin-immunoreactive cell bodies were seen at approximately equal frequency throughout the depths of the sections. All of the galanin cells were NeuN-positive, but most neurons in each lamina were not galanin-immunoreactive (Figure 7). Quantitative analysis with the disector method revealed that galanin-immunoreactive neurons constituted 6.6, 3.1 and 2% of all of the neurons in laminae I, II and III, respectively (Table 2). Since galanin-containing cells are more densely packed in the outer part of lamina II than in its inner part (Figure 2), we estimated the percentage of neurons in the two halves of the lamina that were galanin-immunoreactive, by drawing a line midway between its dorsal and ventral borders. The proportion of neurons in lamina IIo that were galanin-immunoreactive was 5%, while the corresponding value for lamina IIi was 0.4% (Table 2).

**Table 2:** Percentages of neurons in laminae I-III that were galanin immunoreactive

	Number of neurons counted	galanin-immunoreactive cells	% of neurons that were galanin-immunoreactive	% of neurons that are GABA-immunoreactive*	Estimated % of GABA neurons that contain galanin
I	91.3 (80 - 97)	6 (5 - 8)	6.6 (5.2 - 8.2)	24.8	26.4

II	200 (175 - 220)	6 (4 - 8)	3.1 (1.8 - 4.6)	31.3	9.9

IIo	116.7 (99-127)	5.7 (4-7)	5 (3.2-7.1)		

IIi	83.3 (76-96)	0.3 (0-1)	0.4 (0-1.3)		

III	131.7 (104-155)	2.7 (1 - 5)	2 (0.7 - 3.2)	40.2	4.9

In each case the mean values for the 3 animals are shown, with the range in brackets. * The percentages of neurons in each lamina that are GABA-immunoreactive are taken from the data for the L4-5 segmental levels of 3 naïve Sprague-Dawley rats examined by Polgár et al. [1].

### Proportion of GABAergic boutons in laminae I-III that contain galanin

When the sections reacted with antibodies against galanin, CGRP and VGAT were scanned at high magnification each type of immunostaining was found in numerous small structures that presumably corresponded to axonal boutons, while galanin was also seen in cell bodies. CGRP-immunoreactive axons were particularly numerous in laminae I and IIo, but were also found in laminae IIi and III. As reported previously [35], VGAT-immunoreactive boutons were seen at high density throughout laminae I-III. There was no co-localisation of CGRP and VGAT.

**Figure 8:**
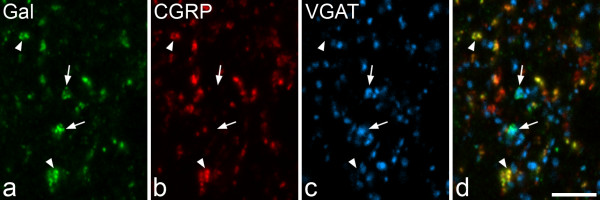
**Co-localisation of galanin with CGRP and VGAT**. **a**, **b**, **c **A confocal image through part of lamina II scanned for galanin (Gal, green), CGRP (red) and VGAT (blue), respectively. **d **A merged image. The small immunoreactive structures correspond to axonal boutons. Several Gal immunoreactive profiles can be seen and most of these are also labelled with the CGRP antibody (two indicated with arrowheads). These appear yellow in **d**. Two of the galanin-positive boutons (arrows) are also immunostained for VGAT. The image was obtained from 2 optical sections at 0.3 μm z-separation. Scale bar = 5 μm.

The majority of the galanin boutons were CGRP-immunoreactive. Many CGRP boutons were also positive for galanin, although CGRP boutons that lacked galanin were also present. Galanin-immunoreactivity was seen in some of the VGAT-positive boutons (Figure 8). The percentages of VGAT boutons in laminae I, II and III that were galanin-immunoreactive were 6.2, 3.3 and 0.7, respectively (Table 3). When lamina II was subdivided into outer and inner halves, the percentages in these were 5.7 and 1.1. The mean of the z-axis lengths for the galanin-positive boutons was 1.14 μm (n = 61), while the corresponding value for the VGAT boutons that lacked galanin was 1.21 μm (n = 1739), and these did not differ significantly (p = 0.09, Mann-Whitney U test). This means that it is unlikely that our sample was biased towards either galanin-positive or galanin-negative boutons among the VGAT population.

**Table 3:** Percentages of VGAT boutons in laminae I-III that were galanin-immunoreactive

Lamina	VGAT boutons analysed	% VGAT boutons Gal+
**I**	600 (100)	**6.2 **(4 - 8.5)

**II**	600 (100)	**3.3 **(3 - 4)

**IIo**	303 (98-103)	**5.7 **(4.1 - 7.1)

**IIi**	297 (97-102)	**1.1 **(0 - 2.2)

**III**	600 (100)	**0.7 **(0 - 1.5)

The second column shows the total number of VGAT boutons from each lamina (or sublamina) that were analysed, with the range per animal (n = 3 rats) shown in brackets. The mean percentages of the VGAT-positive boutons that were galanin-immunoreactive are shown in the third column, with ranges per animal in brackets.

## Discussion

The main findings of this study are: (1) that galanin, which has previously been shown to be expressed only by GABAergic interneurons [26], is present in a different population from those that contain NPY, parvalbumin or nNOS in laminae I-II, although it is co-expressed with nNOS in some lamina III cells, and (2) that the galanin cells constitute around 7% of all of the neurons in lamina I and ~3% of those in lamina II. Since all NPY cells [28,35], and many of those that contain parvalbumin [31,32] and nNOS [33,34], are GABAergic, this indicates that these four compounds can be used to define neurochemically distinct populations of inhibitory interneurons in the superficial dorsal horn.

### Lack of co-localisation of galanin with NPY, nNOS or parvalbumin

Our finding that NPY and galanin show minimal co-existence in neurons in laminae I-III differs from that of Zhang et al. [61], who reported that some galanin-immunoreactive neurons were also NPY-immunoreactive, although they did not determine the frequency of double-labelled cells. We did identify a single lamina II neuron that showed both types of immunoreactivity, but this corresponded to less than 1% of each population, and was therefore an extremely rare event. A significant difference between the experimental protocols of these two studies is that Zhang et al. had applied colchicine to block axoplasmic transport of the peptide from cell bodies. While this increases the sensitivity for detecting peptidergic neurons, it may have direct effects on neuropeptide expression. For example, Cortes et al. [66] reported a dramatic increase in the levels of galanin mRNA in several brain regions after intracerebroventricular administration of colchicine. This could have resulted from up-regulation by cells that normally expressed the peptide, but may also have involved *de novo *synthesis [66]. It is likely that upregulation of galanin or NPY in the superficial dorsal horn following colchicine treatment explains the difference between our results and those of Zhang et al. [61]. It is quite possible that there are neurons in this region that are NPY- or galanin-immunoreactive and also express the other peptide, but at levels that are below our detection threshold. However, even if this is the case, the lack of co-localisation of the two types of immunoreactivity in our experiments provides a useful way of distinguishing two distinct and largely non-overlapping neuronal populations. Using immunogold labelling at the ultrastructural level, Zhang et al. [61] reported occasional co-localisation of galanin and NPY in nerve profiles (presumed axon terminals) in untreated rats. However, in a previous study they had shown co-localisation of galanin, NPY and CGRP [39], and since CGRP is thought to be present exclusively in primary afferent terminals in the dorsal horn, the axons that were labelled for both NPY and galanin are likely to have been primary afferent axons. The almost complete lack of coexistence of NPY and galanin that we found in axonal boutons in laminae I-III further supports the suggestion that the two peptides are present in largely non-overlapping neuronal populations.

The lack of overlap between galanin and parvalbumin populations is consistent with their different locations, since galanin cells are concentrated in laminae I and IIo, while the parvalbumin cells are mainly in lamina IIi and III (Figure 2). We are not aware of any previous studies that have compared the expression of nNOS and galanin among dorsal horn interneurons. However, the lack of co-localisation in laminae I-II is not surprising, because it has been reported that most NADPH diaphorase-positive (nNOS-containing) GABAergic neurons in this region are also enriched with glycine [33], whereas galanin is restricted to those GABAergic neurons that are not glycine-enriched [26]. The presence of nNOS in most of the few galanin neurons that were seen in lamina III suggests that these represent a small but distinctive population of inhibitory interneurons.

Laing et al. [32] reported that NADPH diaphorase activity was not co-localised with either NPY or parvalbumin, and the present results confirm that nNOS is not expressed at detectable levels in neurons that contain either of these compounds. We have previously reported that NPY and parvalbumin do not co-exist in neurons in laminae I-III [32].

### Sub-populations of inhibitory interneurons

The results of the present study show that in the rat L3 segment, galanin-containing cells constitute around 6.6%, 3.1% and 2% of the total neuronal population in lamina I, II and III, respectively. All of these cells are thought to be GABAergic [26], and we have previously reported that 24.8%, 31.3% and 40.2% of neurons in laminae I, II and III at the L4-5 level are GABA-immunoreactive in Sprague-Dawley rats [1]. If we assume that similar proportions of neurons are GABAergic in the L3 segment in Wistar rats, we can estimate the proportion of GABAergic neurons that are galanin-immunoreactive as ~26%, 10% and 5% for lamina I, II and III (Table 2). In a recent study, we quantified NPY-immunoreactive cells (which are also all GABA-immunoreactive [28]) in the L4 segment of Wistar rats and estimated that these account for 23% of the GABAergic neurons in lamina I, 17% of those in lamina II and 9% of those in lamina III. This suggests that cells that contain either galanin or NPY make up around half of the GABAergic neurons in lamina I and a quarter of those in lamina II.

Galanin-containing axons in the dorsal horn are derived from at least two sources: primary afferents and intrinsic dorsal horn neurons [61]. As reported previously [38,39], we found extensive coexistence of galanin and CGRP, which is present in most (if not all) peptidergic primary afferents [67]. Galanin was also found to be co-localised with VGAT, which is restricted to axonal boutons that use GABA and/or glycine as a neurotransmitter [49,50]. The galanin/VGAT-positive profiles are presumably axonal boutons derived from the local galanin interneurons. However, the peptide was only found in around 6% of the VGAT-positive boutons in lamina I and 3% of those in lamina II, even though we estimate that 26% of the GABAergic neurons in lamina I and 10% of those in lamina II contain galanin. One possible explanation for this discrepancy is that a substantial part of the total population of GABAergic boutons in this region does not originate from local neurons, and that this component lacks galanin. Consistent with this, it has been shown that there is a significant descending GABAergic input to the superficial dorsal horn that originates from the caudal ventromedial medulla [68], an area that contains very low levels of galanin mRNA [66]. However, the proportion of GABAergic boutons that are derived from extrinsic sources is not yet known. Another possibility is that the level of expression of galanin is very low in the axons of some cells that contain the peptide at detectable levels in their cell bodies. However, this seems unlikely, as peptide concentrations are generally considerably higher in axonal boutons than in the perikaryal cytoplasm [66]. The most likely explanation is that the galanin cells generate relatively small axonal arbors, and are therefore under-represented among the GABAergic boutons in the superficial dorsal horn. In order to test this, it will be necessary to record from and label individual galanin-containing interneurons, and then examine the extent of their axonal arborisations.

### Functions of galanin-containing neurons

Galanin released by these cells can act on receptors that are expressed by local dorsal horn neurons and by the central terminals of primary afferents. mRNAs for all 3 of the galanin receptors (GalR1-3) have been identified in primary afferents in the dorsal root ganglia, while that for GalR1 is also highly expressed by dorsal horn neurons [69-72]. Galanin acting at spinal levels appears to exert both anti-nociceptive and pro-nociceptive actions, and these are thought to be mediated by GalR1 and GalR2, respectively [73,74]. It has also been suggested that galanin may have neuroprotective and developmental roles mediated by GalR2 [75]. However, the amount of galanin in axons of dorsal horn neurons appears to be lower than that in primary afferents, since dorsal rhizotomy causes a substantial depletion of galanin-immunoreactivity in the superficial laminae [38,57,76]. Therefore release of galanin from intrinsic neurons may be relatively modest, compared to the amount that can be released from primary afferents.

At present, there is little information about the primary afferent input to different types of inhibitory interneuron in the superficial dorsal horn, although it is likely that the galanin-containing cells are innervated by nociceptive afferents since these terminate extensively in laminae I-IIo, where most of the galanin cells are located. Consistent with this suggestion, we have recently found that after injection of capsaicin into the rat hindpaw, ~40% of the galanin-immunoreactive neurons in the medial part of the ipsilateral dorsal horn contain phosphorylated extracellular signal regulated kinases (pERK), whereas this was seen in very few of the nNOS-immunoreactive cells (~2%) and in none of those that contained parvalbumin (S. Tiong, E. Polgár and A.J. Todd, unpublished data). Since pERK is a marker of neuronal activation [77], it is likely that many of the galanin-containing inhibitory interneurons in laminae I-IIo respond to noxious stimulation. This suggests that among the functions that have been proposed for inhibitory interneurons [7], the galanin cells are more likely to have a role in regulating the level of activity of other neurons (including projection cells) in response to noxious stimuli, than in blocking spontaneous activity of projection cells or minimising cross-talk between sensory modalities and preventing tactile allodynia [3,4].

## Conclusions

Laing et al. [32] reported that expression of NPY, nNOS and parvalbumin could be used to identify three different populations of GABAergic interneurons in laminae I-III. The results of the present study show that immunostaining for galanin defines a fourth neurochemical population of inhibitory interneurons in this region. Most of the galanin-containing cells were located in laminae I-IIo, where they accounted for 5-7% of all neurons, with only a small number being present in laminae IIi-III. Based on the results of a previous quantitative analysis of GABA-immunoreactivity [1], we estimate that the galanin cells constitute around 26%, 10% and 5% of the GABAergic interneurons in laminae I, II and III, respectively. However, only ~6% of GABAergic boutons in laminae I-IIo and ~1% of those in IIi-III were galanin-immunoreactive. A possible explanation for this discrepancy is that the galanin cells generate smaller axonal arbors than other GABAergic interneurons in this region.

## List of abbreviations

CGRP: calcitonin gene-related peptide; NADPH: reduced nicotinamide adenine dinucleotide phosphate; NK1r: neurokinin 1 receptor; nNOS: neuronal nitric oxide synthase; NPY: neuropeptide Y; pERK: phosphorylated extracellular signal-regulated kinases; TSA: tyramide signal amplification; VGAT: vesicular GABA transporter;

## Competing interests

The authors declare that they have no competing interests.

## Authors' contributions

SYXT and EP participated in the design of the study and the analysis; JCvK participated in some of the experiments; MW generated the parvalbumin antibody; AJT conceived of the study, participated in design and analysis and drafted the manuscript. All authors contributed to the writing of the manuscript and approved the final version.
